# Case Report: A balloon-based technique to remove a pearl-like embolus out of the coronary artery

**DOI:** 10.3389/fcvm.2023.1086483

**Published:** 2023-04-25

**Authors:** Kaimin Wu, Xiang Chen, Licheng Ding, Bin Wang

**Affiliations:** Department of Cardiology, Xiamen Cardiovascular Hospital, Xiamen University, Xiamen, China

**Keywords:** acute myocardial infarction, coronary embolism, atrial fibrillation, embolus, aspiration thrombectomy

## Abstract

Coronary embolism is considered a rare non-atherosclerotic etiology of acute myocardial infarction, whereas atrial fibrillation is the main etiology of coronary embolism. We report a rare case of a patient with coronary embolism with a specific pearl-like embolus attributed to atrial fibrillation. For this patient, we used a balloon-based technique to successfully remove the embolus from the coronary artery.

## Introduction

**Figure 1:**
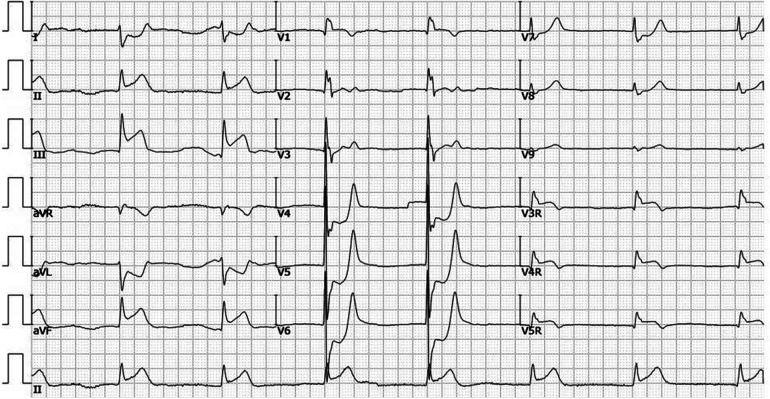
ECG in the ER before the coronary angiogram. ECG showing AF, complete heart block, junctional escape rhythm, and ST-segment elevation in leads II, III, aVF, and V3R-V5R.

A 78-year-old male patient was transferred to the emergency department with a complaint of syncope during defecation. He was reported to have fallen on the floor 4 h before his transfer and to have awakened after a few minutes without chest pain, speech impairment, or limb movement disorder. On admission, his vital signs were as follows: pulse rate, 42 beats/min; blood pressure, 84/44 mmHg; and respiratory rate, 16 breaths/min. A physical examination revealed no neurological signs and grade V/V muscle strength. An electrocardiogram showed atrial fibrillation (AF), complete heart block, junctional escape rhythm, and ST-segment elevation in leads II, III, aVF, and V3R-V5R (Figure 1). Laboratory tests showed an initial troponin T of 6,938 ng/L; total cholesterol, 142 mg/dl; low-density lipoprotein cholesterol, 82 mg/dl; high-density lipoprotein cholesterol, 43 mg/dl; and triglycerides, 36 mg/dl. Transthoracic echocardiography revealed a mild hypokinesis in the inferoposterior wall, moderate mitral regurgitation, and no intracardiac or left atrial thrombus (left ventricular ejection fraction of 49%). The patient had a previous medical history of atrial fibrillation for more than 30 years but was not taking any anticoagulant drugs. He had no history of smoking, hypertension, or diabetes mellitus.

A diagnosis of acute inferior and right ventricular ST-segment elevation myocardial infarction was made. Aspirin (300 mg) and ticagrelor (180 mg) were given. A temporary pacemaker (frequency 60 bpm, voltage 5 mv) was implanted to maintain a normal heart rate through the right femoral vein.

**Figure 2:**
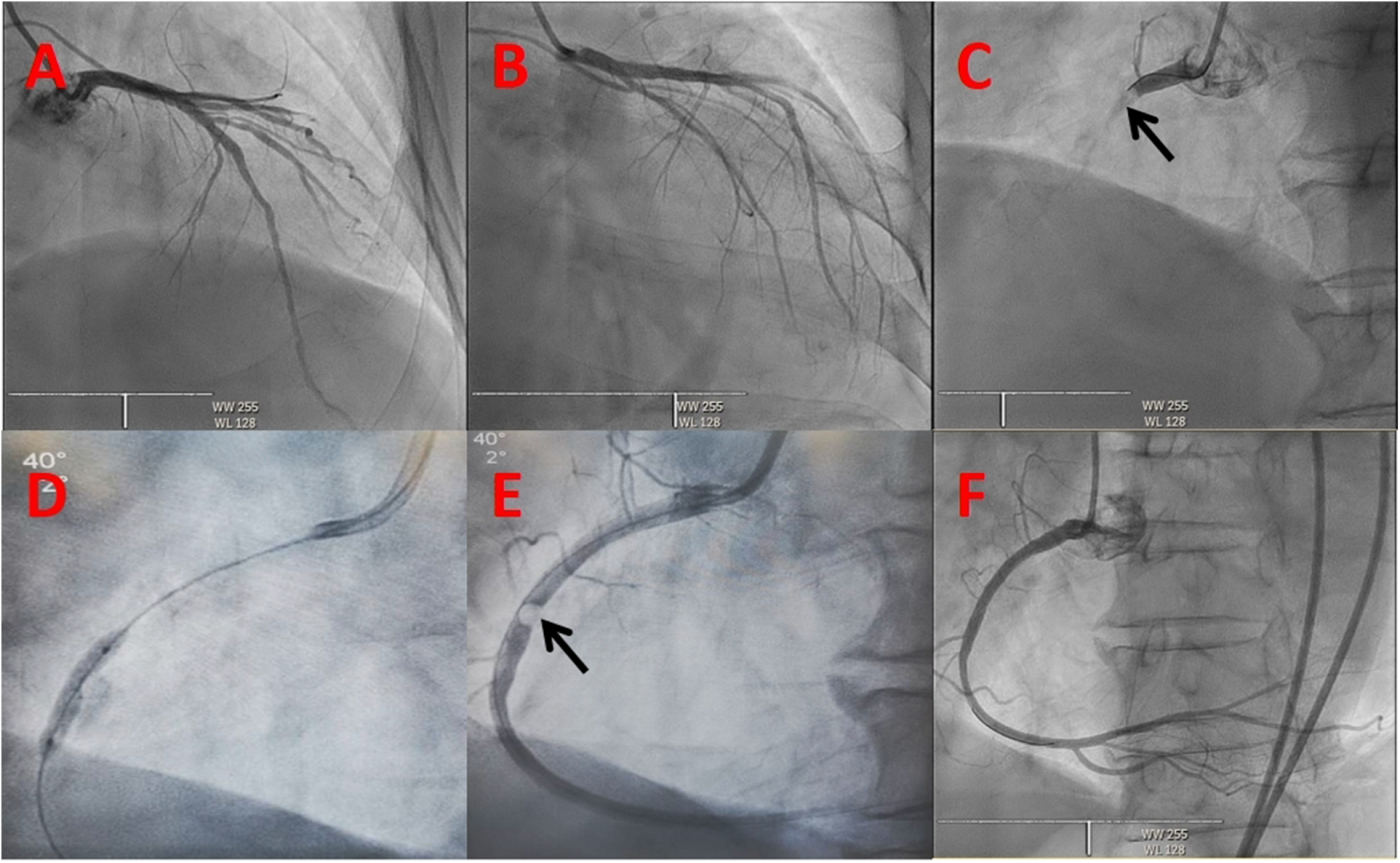
Coronary angiogram. The initial view of the coronary angiogram in (**A–C**) revealing a normal left coronary system and an embolism in the proximal right coronary artery (dark arrow): (**A**) Cranial 30, (**B**) RAO 30 + Caudal 30, (**C**) LAO 40. (**D**) Hourglass sign of balloon angioplasty. (**E**) Embolus in the proximal right coronary artery (dark arrow). (**F**) Right coronary angiogram showing TIMI 3 blood flow after the intervention.

**Figure 3:**
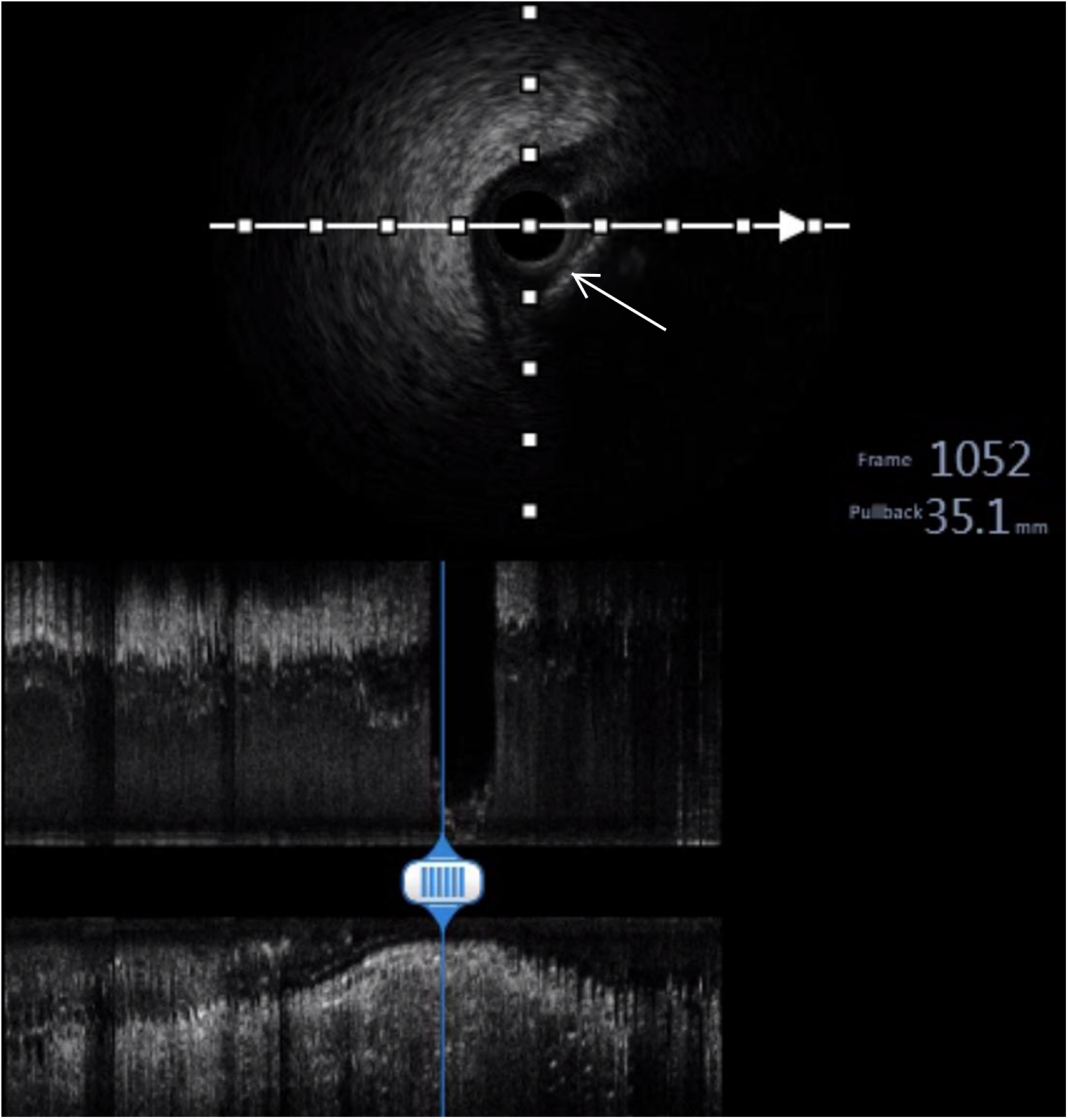
Intravenous ultrasound (IVUS). IVUS showing calcification and posterior echo attenuation in the middle RCA (white arrow).

Unfractionated heparin was given by loading 6,000 IU intravenously and infusing with 1,000 IU per hour during the procedure to maintain the activated coagulation time greater than 300 s. An emergency coronary angiogram showed a total occlusion of the proximal right coronary artery (RCA) with a globular filling defect without any collateral vessels from the left coronary system (Figure 2). A 6 Fr JR 4.0 guiding catheter (Teruma, Japan) was engaged in the RCA. Run-through wire (Teruma, Japan) and Sion wire (ASAHI, Japan) failed to pass through the lesion. We considered that the occlusion was not a common thrombotic lesion and attempted to open the lesion using Fielder XT (Abbott, USA) wire under the Finecross microcatheter (Teruma, Japan). With careful manipulation, the wire was advanced to the distal end of the RCA through the occlusion. A 1.5 × 15 mm Maverick balloon (Boston Scientific, USA) was successfully passed through the lesion and was inflated to 6 atm, but the coronary angiogram showed no blood flow in the RCA. The balloon was converted to a 2.0 × 20 mm Maverick balloon and inflated to 6 atm, but again, it did not yield the desired results. We then performed aspiration thrombectomy using Rebirth (Goodman, Japan). But no thrombus was aspirated. Then, we used a Sprinter balloon of different sizes (2.0 × 20 mm and 2.5 × 15 mm) (Medtronic, USA), which successively dilated the lesion, and an hourglass sign was revealed (Figure 2). The blood flow recovered to TIMI 1 and the embolus ran deeper into the RCA (Figure 2). To make the characteristics of the embolus clear, we decided to perform intravenous ultrasound (IVUS) (Boston Scientific, USA). IVUS showed calcification and posterior echo attenuation in the mid-RCA (Figure 3). We realized that the embolus was hard and calcified, and the usual methods would not work. The option of emergency coronary artery bypass grafting was considered. But the patient's hemodynamics were still unstable, and he suffered ventricular fibrillation several times during the procedure. The cardiac surgeon reasoned that the mobility of the embolus in the RCA rendered it difficult to be detected accurately and that the surgical risk was too high. The patient's family also did not favor surgery. After a detailed discussion, we decided to use a balloon-based technique to extract the embolus from the coronary artery. First, we would use a balloon to pass through the embolus to the distal portion of the RCA. Second, the balloon would be inflated to low pressure and then be pulled backward to bring the embolus together out of the coronary artery. Finally, we would perform selective organ angiography to locate the escaped culprit embolus and prevent embolization to the major related organs, i.e., brain, intestines, and kidneys.

**Figure 4:**
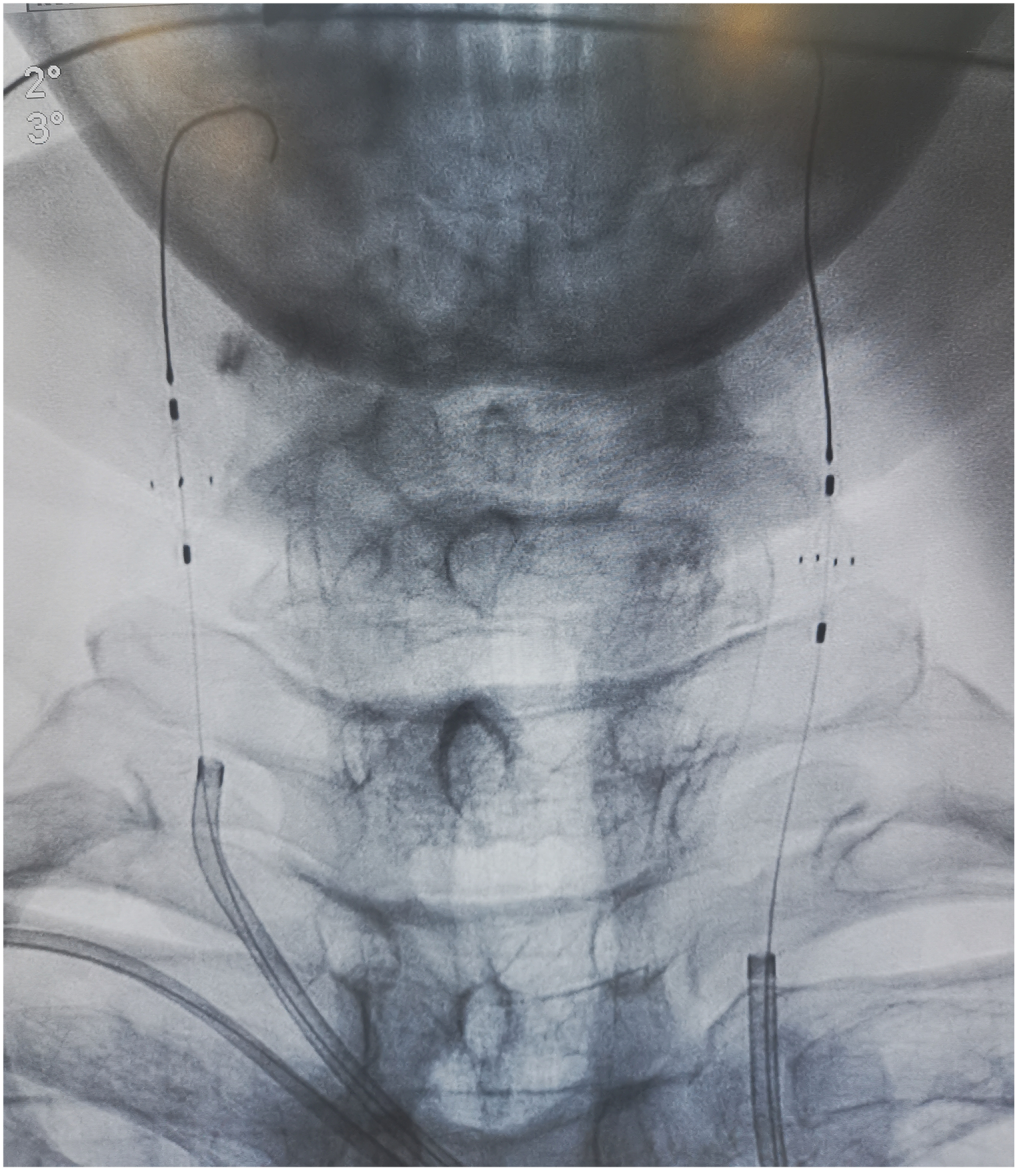
Angioguard umbrellas in both carotid arteries.

**Figure 5:**
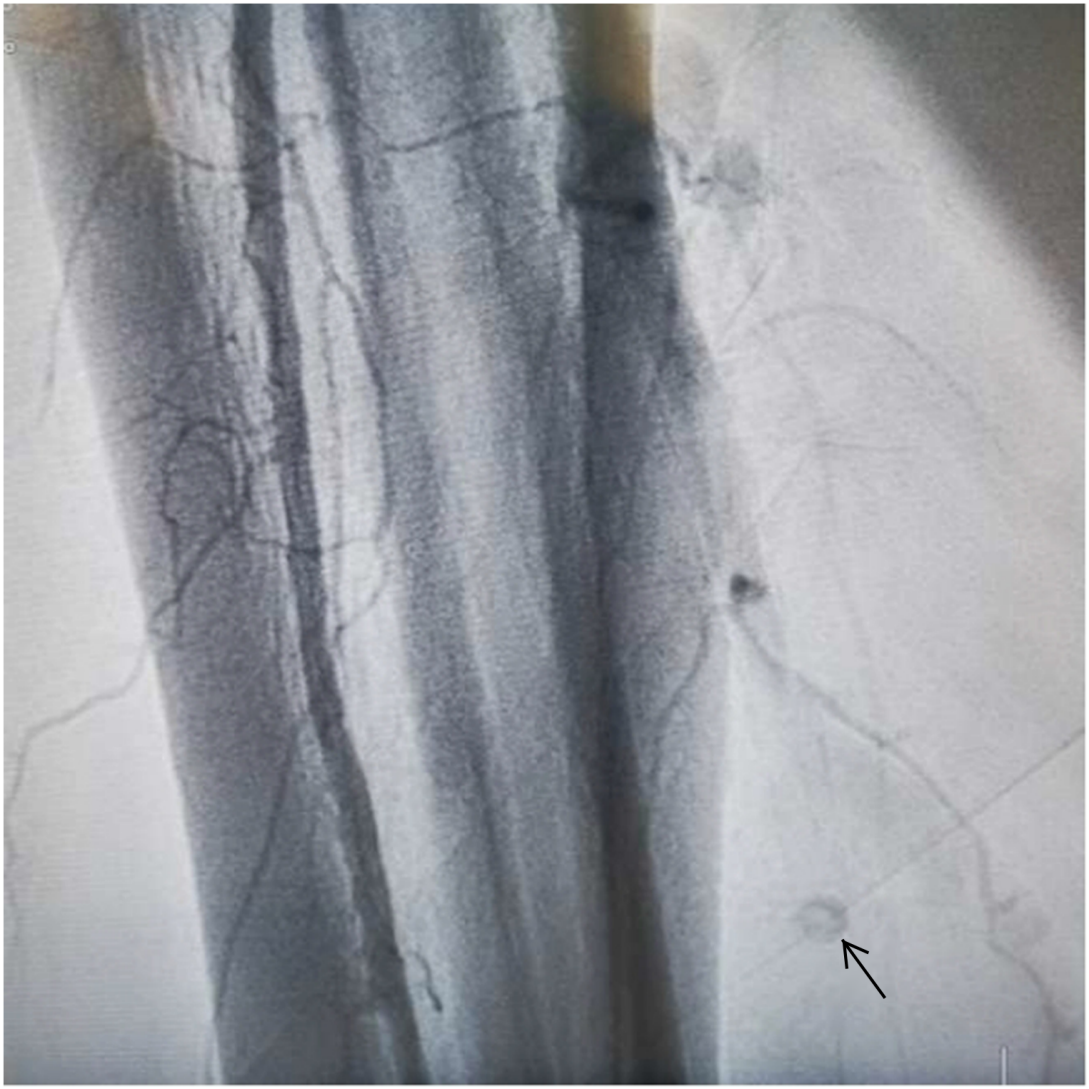
Lower limb artery angiography showing a filling defect (dark arrow) in the right peroneal artery.

**Figure 6:**
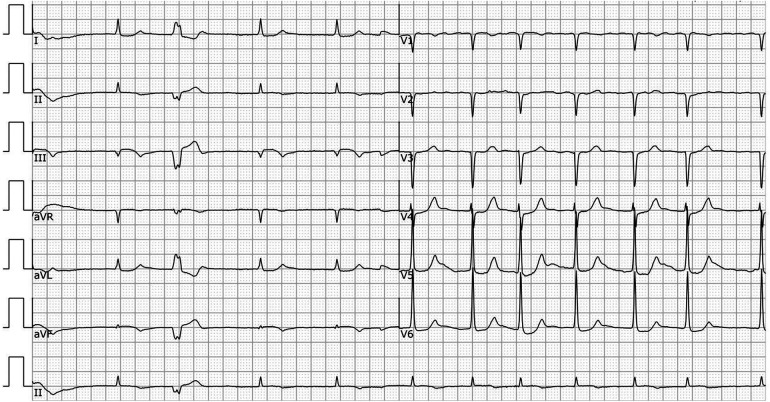
ECG after the procedure. ECG showing AF, and premature ventricular beats.

An 8-Fr short dilator was inserted through both femoral arteries, and Angioguard (6 mm) umbrellas (Cordis, USA) were inserted in both carotid arteries through an 8-Fr MPA catheter (Cordis, USA) (Figure 4). A 2.5 × 15 mm Sprinter balloon was successfully passed through the lesion and was inflated to 4 atm. Then, we dragged the balloon and the embolus together out of the coronary artery carefully and slowly. A subsequent coronary angiogram in the RCA showed that blood flow had recovered to TIMI 3. Further, we performed a cerebral artery, superior mesenteric artery, and lower limb artery angiogram to confirm the escape site of this embolus after the removal procedure with vital organ protection. Fortunately, we found that the embolus was brought to the right peroneal artery (Figure 5). The patient did not feel any lower limb pain, and the pulse of the dorsalis pedis artery was palpable. Finally, we completed this tough coronary procedure, and the total time consumed was 4 h and 53 min. The patient was then safely transferred to the intensive care unit. An electrocardiogram after the procedure revealed that the ST segment had returned to baseline and no significant Q wave was detected in the inferior leads (Figure 6).

**Figure 7:**
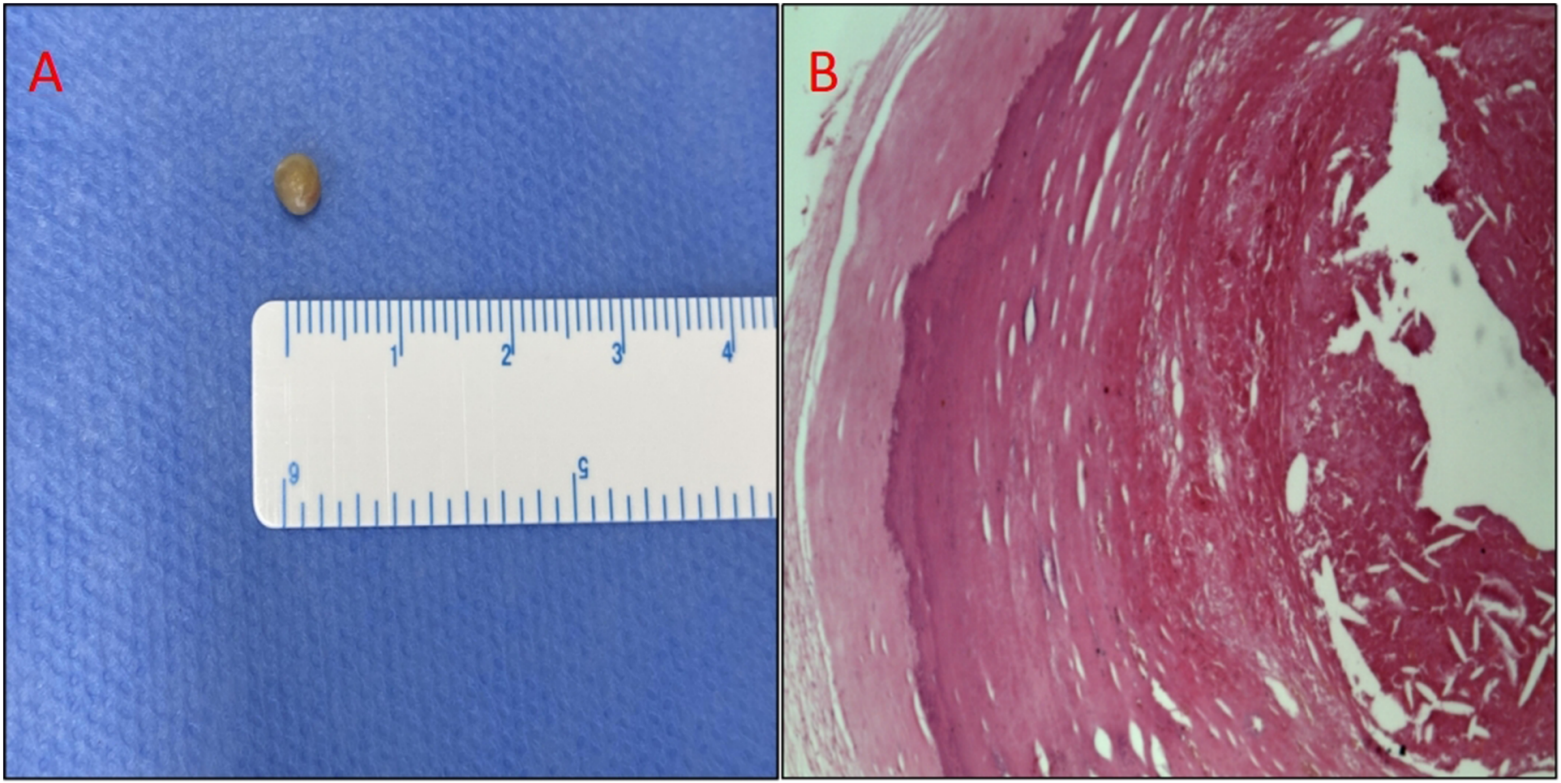
(**A**) Photograph of the retrieved embolus: gray globular tissue with a diameter of 4 mm. (**B**) Photomicrograph of the embolus. Hematoxylin and eosin staining. At the center of the embolus is fibrinoid necrosis with a few mixed red blood cells, and the surrounding is a thick layer of fibrotic tissue with hyaline degeneration and peripheral calcification.

The next day, we decided to remove the embolus from the body with the help of a vascular surgeon in order to avoid the risk of the patient contracting lower limb ischemia. We used the same method to drag the embolus from the right peroneal artery to the right femoral artery and then pulled it out of the body by incising the femoral artery. The culprit turned out to be a hard gray pearl-like embolus. Further histopathological examination revealed the organized and calcified nature of the embolus. Fibrinoid necrosis developed at the center of the embolus with a few mixed red blood cells, and surrounding it was a thick layer of fibrotic tissue with hyaline degeneration and peripheral calcification (Figure 7).

After the procedure, the patient did not experience recurrent myocardial infarction, but he suffered congestive heart failure and gastrointestinal bleeding (melena) in the hospital. With guideline-directed medical treatment, he showed recovery and was discharged one month later with an antithrombotic therapy of rivaroxaban 15 mg qd + clopidogrel 75 mg qd. He recovered well afterward and remained in NYHA class I at month 3 of follow-up.

Coronary embolism is an under-recognized etiology of AMI with the potential to yield significant morbidity and mortality rates. A retrospective study found that the prevalence rate of coronary embolism in patients with AMI was 2.9% and that AF was the most important cause (1). Aspiration thrombectomy remains the mainstay of treatment for coronary embolism (2, 3)^.^ However, in the case of a more organized and calcified embolus, as in this case, aspiration thrombectomy may not be effective, and other unconventional techniques may be needed to be used. IVUS is very important because it can help us clearly define the characteristics of the embolus. In this case, the technique of dragging the embolus out of the coronary artery using a balloon inflated with low pressure may be an effective choice. However, it should be noted that the protection of the brain and other major related organs is very important because it will be catastrophic if the embolus causes a stroke or other systematic embolisms.

## Data Availability

The original contributions presented in the study are included in the article/**Supplementary Material**, further inquiries can be directed to the corresponding author.
